# Intermittent ELF-MF Induce an Amplitude-Window Effect on Umbilical Cord Blood Lymphocytes

**DOI:** 10.3390/ijms232214391

**Published:** 2022-11-19

**Authors:** Lucián Zastko, Leonardo Makinistian, Andrea Tvarožná, Igor Belyaev

**Affiliations:** 1 Department of Radiobiology, Cancer Research Institute, Biomedical Research Center, Slovak Academy of Sciences, University Science Park for Biomedicine, 845 05 Bratislava, Slovakia; 2 Department of Laboratory Medicine, Faculty of Health Care, Catholic University in Ružomberok, 034 01 Ružomberok, Slovakia; 3 Department of Physics and Instituto de Física Aplicada (INFAP), Universidad Nacional de San Luis-CONICET, San Luis CP5700, Argentina

**Keywords:** apoptosis, viability, inhomogeneous magnetic fields, reactive oxygen species

## Abstract

In a previous study of the effects of intermittent extremely low frequency (ELF) magnetic fields (MF) on umbilical cord blood lymphocytes (UCBL), we evaluated MF amplitudes between 6 µT and 24 µT and found an effect only for those below 13 µT. This suggested the existence of an amplitude window. In this brief communication, we further tested this hypothesis. UCBLs from healthy newborns were isolated and exposed for 72 h to an intermittent ELF-MF (triangular, 7.8 Hz, 250 s ON/250 s OFF) with 6 different amplitudes between 3 µT and 12 µT, utilizing an oblong coil. Percentage of viable, early apoptotic (EA), and late apoptotic/necrotic (LAN) cells were determined by flow cytometry. Moreover, reactive oxygen species (ROS) were determined at 1 h and 3 h of the exposure. Like in our previous work, neither EA, nor LAN, nor ROS were statistically significantly affected by the intermittent ELF-MF. However, the percentage of viable cells was decreased by exposure to the fields with intensities of 6.5 µT and 12 µT (*p* < 0.05; and *p* = 0.057 for 8.5 µT). ELF-MF decreased the percentage of viable cells for fields down to 6.5 µT, but not for 5 µT, 4 µT, or 3 µT. Combined with our previous findings, the results reported here indicate an amplitude window effect between 6 µT and 13 µT. The obtained data are in line with a notion of amplitude and frequency windows, which request scanning of both amplitude and frequency while studying the ELF-MF effects.

## 1. Introduction

While interest on the influence of extremely low frequency (ELF) magnetic fields (MF) on blood cells goes as far back as the 1950s [1], there was a remarkable rise in the 1990s due to the association found between wire configuration codes of electrical energy lines and the incidence of childhood leukemia reported in the pioneering work by Wertheimer and Leeper [2]. That work plus a remarkable body of others that followed it led the International Agency for Research on Cancer to classify ELF-MF as possibly carcinogenic to humans (Group 2B) in 2002 [3] (p. 338). Since then, a number of reports have continued to study the issue from an epidemiological point of view [4,5,6], including pooled analyses [7,8,9], but also with in vivo and in vitro experiments (also see updated reviews [10,11]).

In in vitro experiments, MF (50 Hz sine, 1 mT, intermittent 5 min on/10 min off) were shown to affect the robustness of epigenetic programming during granulopoiesis in leukemic Jurkat cells and in human CD34+ hematopoietic stem cells undergoing in vitro differentiation into the neutrophilic lineage [12]. Uckun et al. exposed B-cells to 60 Hz (0.1 mT) MF for 1–30 min to observe activation of protein Tyr kinase Lyn, as well as of the serine/threonine kinase protein kinase C (PKC) [13]. While a 5 min exposure of Jurkat cells to 50 Hz (0.10 mT) also resulted in increased Tyr kinase Lck [14], no effect was seen on the activity of Bruton’s tyrosine kinase in DT40 lymphoma B cells upon exposure to 0.1 mT 60 Hz for 15 s–5 min [15]. Kapri-pardes et al. [16] reported 2–3-fold increases in ERK1/2 phosphorylation in Jurkat (and also other cell lines) after a 5 min exposure (1 mT, 50 Hz, 5 min/1 min on/off periods). A novel mouse model was developed to study the role of ELF-MF in childhood leukemia as part of the ARIMMORA project. This model did not show a statistically significant susceptibility to ELF-MF in a pilot study, but represents a key resource for future in vivo experimentation [17,18].

While the aforementioned studies focused on 50 Hz and 60 Hz fields, clearly motivated for their use in the generation and distribution of electrical power, the effects of static magnetic field (SMF, alone and in combination with other agents) on lymphocytes have also been investigated [19,20,21]. Further departing from the punctual concern about 50 Hz and 60 Hz fields, other frequencies have been explored. For instance, the anomalous viscosity time dependence (AVTD) of lymphocytes lysates was measured after a 20 min exposure to 21 µT_rms_ fields, 1–12 Hz and 52–65 Hz, showing differences between frequencies and also between different donors [22]. Teratological studies were carried out on rats, where a slight but significant decrease in the lymphocyte count was observed (sawtooth waveform, 66 µT, 56 µs period) [23]. Inhibition of concanavalin-induced Ca^2+^ influx in rats’ thymic lymphocytes was observed at 16 Hz, 42.1 µT [24]. Moreover, in the opposite end of evaluating their possibly detrimental effects, in vivo experiments with MF (rotating, 0.2 T, 4 Hz) have been suggested to improve the autoimmune encephalomyelitis by promotion of T-cell peripheral accumulation [25].

It is clear from the literature that, not only have the questions about the possible oncogenicity of 50–60 Hz not been answered, but also further lines of research have been added to them. Considering possible biophysical mechanisms, both frequency and amplitude windows were predicted and validated experimentally [26]. The issue of frequency and amplitude windows seems to be a key aspect of the complex phenomena at hand. In this brief communication, we aimed to test the hypotheses we put forward in our previous study, where the existence of an amplitude-window was suggested by our findings in experiments of exposure of umbilical cord blood lymphocytes (UCBLs) to intermittent ELF-MF [27]. While our results presented here confirm such hypothesis, they warrant cautious consideration and point to the need for further research.

## 2. Results

**Figure 1:**
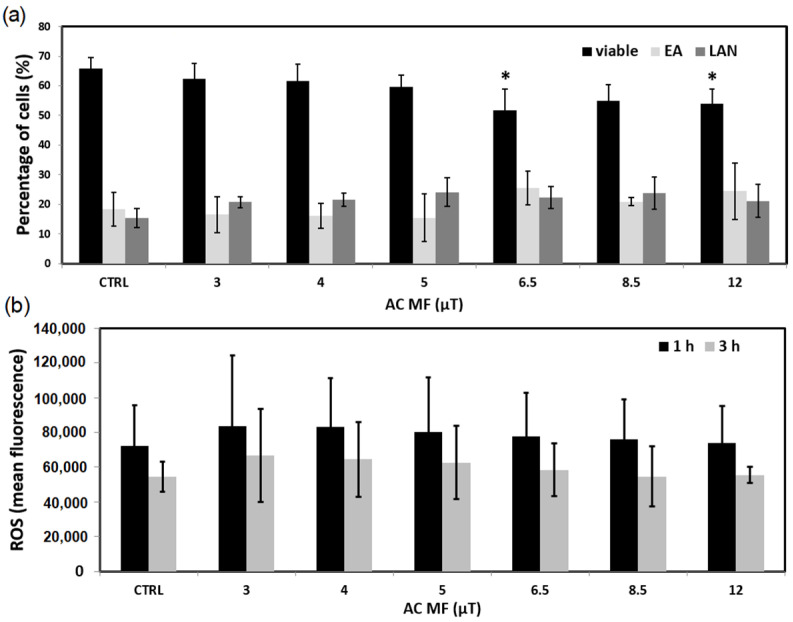
Exposure of UCBL to the 7.8tAM waveform during 72 h (N = 3). (**a**) Percentages of viable, early apoptotic (EA) and late apoptotic/necrotic (LAN) cells. (**b**) ROS production. All alternating current (AC) MF field values are peak values. * *p* < 0.05. Bars denote standard deviation (SD).

Figure 1a shows the percentage of viable, early apoptotic (EA), and late apoptotic/necrotic (LAN) cells determined by flow cytometry. A tendency of viability to decrease can be observed towards the highest MF. In fact, there were statistically significant values for 6.5 µT and 12 µT, where, compared to control, they showed a 21.7% and 18.0% decrease, respectively. In contrast, no effects were observed in the production of ROS (Figure 1b) at either 1 h or 3 h of exposure (for any of the MF amplitudes).

**Figure 2:**
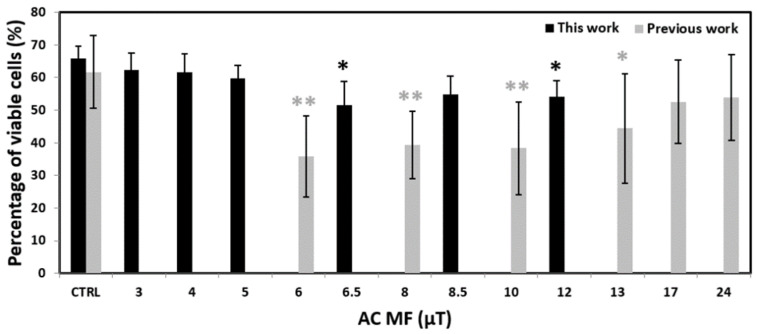
Percentage of viable cells after a 72 h exposure of UCBLs to the waveform 7.8tAM: our results from a previous work [27] combined with those of the present work. Experiments were done six times in our previous work (N = 6) while in triplicate in this work (N = 3). All MF values are peak values. * *p* < 0.05, ** *p* < 0.01. Bars denote standard deviation (SD).

The sole decrease of the viability by around 20% for 6.5 µT and 12 µT might seem a marginal result that, nevertheless, leaves the question of what would be the effect for greater MF intensities (above 12 µT). However, if pooled together with our previous experiments [27], performed with all the same parameters, except by the intensity of the current injected to the coil (we used 50 mA_peak_ here, while 100 mA_peak_ before), then that question is answered. Indeed, Figure 2 shows the same data of Figure 1a for viability along with our previous data, which were obtained for the 6–24 µT range of MF intensities, that is, overlapping between 6 and 12 µT with those explored here, but then going further up to 24 µT. If taken together, the experiments indicate an amplitude window in the 6–13 µT range.

## 3. Discussion

According to Markov [28], the term “biological window” was coined by S. M. Bawin and W. Ross Adey in their 1976 article on the efflux of Ca^2+^ from chick and cat brain tissue [29] upon the exposure to weak low frequency electric fields. In their work, the authors identified both frequency and amplitude windows, i.e., an effect was seen within certain ranges of those parameters, but not outside of them. Since then, other studies have reported such windows. For instance, Aarholt studied the influence of 50 Hz and 16.66 Hz square waveform magnetic fields in the population (0–22 mT) [30] and the rate of β-galactosidase synthesis (200–700 µT) [31] of *E. coli* cultures. Another remarkable example of amplitude windows is given in the maximum relative viscosity (MRV) index of *E. coli* lysates presented by Binhi et al. [32], where the authors also provided a quantum mechanical model to account for their experimental findings. In their work, the MRV displays positive, negative, or null changes when compared to control, depending on the SMF applied (0–100 µT). Another outstanding example is found in the experimental work by Blackman et al. [33] on the effect of parallel direct current (DC) + AC on nerve growth factor-induced neurite outgrowth in PC-12 cells, where not only did they find noticeable AC-field amplitude windows, but they also showed their consistency with the ion parametric resonance (IPR) model of interaction [34]. More recently, working with SMF, Van Huizen et al. [35] showed positive, negative, and null changes in planarian regeneration within the 0–600 µT range. A further example is that of SMF (0–188 µT) applied to *Arabidopsis thaliana*, which produced significant changes in several genes’ expressions [36]. Overall, these studies show that field changes of a few µT (in some experiments) or in the orders of 10–100 µT (in others) can be enough to enter (or exit) an amplitude window, which is in line with our findings. Furthermore, even smaller fields, in the order of hundreds of nT, were exhaustively explored in the exposure of mice to a combination of DC and AC fields, resulting in the inhibition of Ehrlich ascites carcinoma progression for very specific amplitude windows [37]. Of particular relevance are the frequency and amplitude windows reported by Lindström et al. [38] on the intracellular calcium concentration in Jurkat cells. The authors studied the 5–100 Hz frequency range and amplitudes between 0.04 and 0.3 mT, with the highest effects at 50 Hz and 0.15 mT (with no further increase for fields up to 0.3 mT).

The above-retrieved examples, plus the results presented here, widen the perspective from which to interpret reports performed at a single amplitude and frequency. For instance, Toda et al. [39] reported remarkable effects of a series of 10 µT (of notice: just within our amplitude window), 4 ms pulses at 1–8 Hz on mitochondria, finding that they reduced mitochondrial mass to 70% and the mitochondrial electron transport chain (ETC) complex II activity to 88%, which ultimately induced mitochondrial rejuvenation. Further, the authors prompted that such exposure could be studied for the treatment of pathologies such as Parkinson’s disease. In the context of our findings, it would be pertinent to evaluate whether their reported effects also present an amplitude window. Beyond the discovery of thresholds, this could bring about an optimization of the field parameters within such windows. Moreover, if the use on humans is in discussion, the relevance (or lack of it) of the background SMF should be evaluated. The same is relevant regarding our findings. We performed our experiments at a static background MF of 3.7 µT and, *a priori*, our results could have depended on that background conditions.

The fact that we did not find an effect in ROS also deserves some reflection. We evaluated this endpoint at 1 h and 3 h of exposure. It could be that there were effects close to the beginning of the exposure but they were compensated by the cellular response to an imbalance of those molecular species. In addition, it could be that, having adapted to the MF over the duration of the exposure, the end of it caused an effect in ROS that, nevertheless, was compensated in less than one hour. These kinds of homeostatic responses can readily be described by a black-box approach consisting of a feedback loop with a gain and a delay, meaning that the response of the cell to a perturbation (e.g., an increase in ROS) triggers a compensating action that is proportional (hence, the gain) to the perturbation but that takes place with a certain delay after the perturbation. This model was discussed in depth by Barnes and Kandala [40], and has the appealing feature of predicting frequency but also amplitude windows of response, such as the one we have found. A further addition of complexity is given by the fact that timing of the perturbation with respect to internal oscillations can either increase or decrease their amplitude. In order to study this sensibility to timing, the use of synchronized cultures [41] or fluorescence microscopy (simultaneous to exposure) [42] could be considered. It is worth noting that given the generality of this gain/delay feedback loop model, it does not only apply to our results on ROS, but also to the amplitude window found between 6 and 13 µT. As to why the window is “flat” (i.e., no differences found *inside* the window), it could be due to a relatively high variability of our determinations as compared to the observed size effect, which could be masking some subtle “intra-window” variation.

Furthermore, we notice that background RF fields were different at the control and exposure sites (200 nT_rms_ and 90 nT_rms_, respectively). While this difference is not to be disregarded (ideally control and exposed cells should be exposed to exactly the same background), the fact that we have significant differences within the exposed plate (which even included unaffected rows) sustains our interpretation of the AC amplitude being the cause for those differences.

Moreover, the angle between the static and AC field could be important here, as it has been shown by Naarala et al. in proliferation studies were rotating the AC field by 90° changed the results substantially [43]. Testing the relevance of the AC/DC angle certainly deserves more research in the future, which we could attempt by placing the oblong coils in the center of 3D Helmholtz coils, in order to fully control the background field [35].

The interrelation between static and ELF-MF have been discussed for decades [26], and it still remains poorly understood. The link of SMF and RF fields (which on their own deserve careful experimentation [44] not only in vitro and in vivo, but also in humans, where, e.g., they were reported to alter the heart rate variability of adolescents [45]) has received relatively less attention, although remarkable findings have been reported in magnetoreception [46,47,48]. An even less explored arena is the simultaneous combination of static, ELF, and RF fields which are, in fact, the more common condition to which general population is exposed to. In this regard, exposure from mobile phones is, perhaps, the worthiest of being investigated, due to their massive use, including children and adolescents [45]. Overall, either investigating SMF, ELF MF, or RF fields alone or in combination with each other, the sum of an extensive background of both experimental and theoretical reports make it sensible to expect amplitude and/or frequency windows [26].

## 4. Materials and Methods

### 4.1. Cell Culture

This study was approved by the Ethics Committee of Children’s Hospital in Bratislava, Slovakia. Mononuclear cells (MNC) were extracted as previously described [49] from UCB of healthy newborns after full-term pregnancies, frozen in liquid nitrogen, and provided by Dr. M. Kubes, Eurocord-Slovakia, Bratislava, Slovakia.

**Figure 3:**
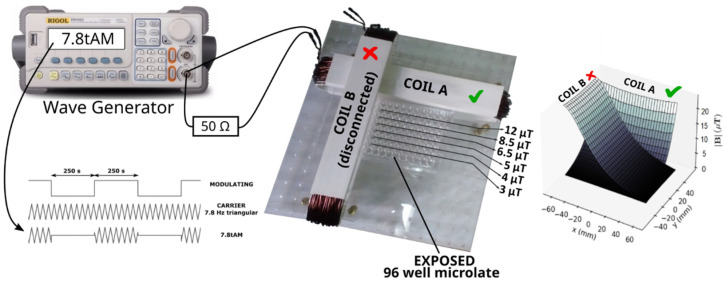
Scheme of the experimental set up. A 96-well microplate is exposed to a magnetic field generated by injecting a 7.8tAM waveform to one of the coils (COIL A) of the system of orthogonal coils. An oscilloscope (not shown in the picture) is hooked to a 50 Ω resistor connected in series with COIL A. The different rows of the microplate are exposed to different magnetic fields between 3 µT and 12 µT. To the right, a 3D representation of the fields that can be produced by the coils (in our experiments COIL B was disconnected, so no field was generated by it).

For each experiment, 3–5 × 10^7^ of frozen UCB MNC cells from individual newborns were briefly thawed in a water bath and diluted in 10 mL of thawing medium containing 4.5 mL of Hanks’ Balanced Salt Solution (HBSS) medium (Gibco, Life Technologies, Renfrew, UK), 1 mL of 1 mg/mL DNase I (Sigma-Aldrich, St. Louis, MO, USA), and 4.5 mL of Roswell Park Memorial Institute (RPMI) 1640 medium with L-glutamine and 4-(2-hydroxyethyl)-1-piperazineethane-sulfonic acid (Hepes) (PAA Laboratories, Pasching, Austria). Adherent monocytes were excluded after 2 h incubation of cells in 20 mL of basal medium (BM): RPMI 1640 medium, supplemented with 10% fetal bovine serum (FBS), 100 IU/mL penicillin, and 100 mg/mL streptomycin (Gibco, Invitrogen, Darmstadt, Germany). Viability of remaining UCBLs was not less than 95% as determined by the Trypan blue exclusion assay. UCBLs were spun down at 100 g, diluted in BM to cell density of 1 × 10^6^/mL and then 200 µL of cell suspension were added to each well of two 96-well plates: one labeled as “exposed” and one as “control”. The former one was placed in the system of oblong coils [50] (Figure 3) previously used in our proliferation study with cancer cell lines [51], and the latter one was placed at the center of a plastic shelf in the same NB-203XL humidified incubator (N-BIOTEK, Bucheon, Republic of Korea), at 37 °C and 5% CO_2_. The control plate was 24.4 cm above the exposed one; at this distance, no fields from the MF-generating coil could be detected.

### 4.2. Exposure to Intermittent ELF-MF

Here, we used the system developed and described in full detail in [50]. Briefly, it consists of two oblong coils put at a right angle to each other, Figure 3 (center). The geometry and size of the system is such that a standard 96-well microplate can be placed close to the coils and, when they are injected with a current, magnetic fields are generated with lines of constant intensity parallel/perpendicular to the microplate rows/columns, Figure 3 (right).

Two plates (“control” and “exposed”) were filled completely with a 12-channel pipette using the technique of reverse pipetting to enhance repeatability [52]. Immediately after exposure, 8 wells from the center of the control plate (wells D5–D8 and E5–E8) were transferred to a 15 mL Falcon tube labeled as “CTRL” to be further processed as described above. UCBL in periphery wells of the exposed plate were not included in any subsequent analysis, due to the well-known risk of faster evaporation in these wells (so called, “edge effect”). Hence, only rows from B to G and columns from 2 to 11 were analyzed (cells in rows A and H, and in columns 1 and 12 did not enter the assays). Cells from ten wells of row B (columns 2 to 11), which were all subjected to the same MF strength (12 µT, see Figure 3) were pooled together and transferred to a Falcon tube labeled as “12 µT”. The procedure was repeated for rows C through G with the corresponding labels: “8.5 µT” (row C), “6.5 µT” (row D), “5 µT” (row E), “4 µT” (row F), and “3 µT” (row G), Figure 3. The reason to pool together cells from the ten wells of each row B-G (in addition to them being subjected to the same exposure) was to have enough cells for the cytometry assay (see below). Cells from the seven obtained tubes (1 control and 6 exposed) underwent the assays described below. A total of 3 independent experiments were carried out.

In this study, no current was injected to the coil parallel to the columns of the exposed plate, whereas an alternated current (AC) was injected to the coil parallel to the rows of the exposed plate. The AC current was a 50 mA_peak_ 7.8 Hz symmetric triangular turned ON and OFF for equal intervals of 250 s (Figure 3); we called this waveform “7.8tAM” here, same as in our previous studies [27,51]. During the intervals with the signal OFF, the current injected to the coil decreased to 1% of its peak value (which was the minimum deliverable by our wave generator). In other words, the peak value of the injected current alternated intermittently between 50 mA_peak_ and 0.5 mA_peak_, making the maximum AC field go between 12 µT_peak_ (“ON”) and 120 nT_peak_ (“OFF”). Of note, is that the value of 120 nT_peak_ is of the order of magnitude of the background AC field (90–200 nT_rms_ so, roughly, 130–280 nT_peak_, see below). The geometry of the exposure system implies that the wells of each row of the plate are exposed to the same ELF-MF, which justifies pooling cells from each row together for further analysis. The AC current was generated by a DG1022 arbitrary wave generator (Rigol, Beaverton, OR, USA) and monitored with a DS1052E digital oscilloscope (Rigol) hooked to a 50 Ω resistor in series with the AC coil. According to the thorough characterization of our coils [50], they produce a temperature increase of 0.2 °C at well A1 upon the injection of a current of 600 mA DC. Therefore, given that Joule heating is proportional to the square of the root-mean-square (RMS) current, the estimated temperature increase in our experiments with triangular 100 mA_peak_ AC current at 50% duty cycle was lower than 10^−3^ °C. The calculated increase of temperature was far below the error of ordinary microthermocouples and infrared thermometers (0.1–0.2 °C) which was confirmed by our measurements inside cell media with applied MFs. Hence, we discarded temperature as a confounding variable. The electric fields (E-fields) induced by the MF generated by our coil were of the order of 10^−9^–10^−8^ V/m [27]. Of note, this value is 11–12 orders of magnitude lower than the E-field across a 10 nm thick cell membrane with a typical transmembrane potential of 50 mV, which turns out to be 62.5 × 10^3^ V/m (assuming an electrical permeability of ~ 80 present in a pore or an ion channel). Because the threshold for an E-field to elicit a biological effect was estimated to be around 10^−4^ V/m [42,53], we followed the standard practice of reporting our results as a function of the MF and not their induced E-fields. The background AC field, measured with a TM-192 3-axis magnetometer (Tenmars, Taipei, Taiwan), was of ~90 nT_rms_ and ~200 nT_rms_ at the exposure and control sites, respectively. The radiofrequency (RF) background radiation, measured with a TM-196 RF 3-axis field strength meter (Tenmars, Taipei, Taiwan), was the same at both sites: E = 0.5 mV/m, H = 1.378 µA/m, S = 0.001 mW/cm². The DC background field was assessed with an HCM5883L 3-axis magnetometer (Honeywell, Morris plains, NJ, USA). It was homogeneous throughout the exposed plate (within 92%) and in average equal to 3.7 µT (in order to reduce external fields, we lined the inner walls of our incubator with 0.1 mm thick mu-metal). The angle between the background DC fields and the AC MF generated by the coil ranged between 91° and 104° throughout the plate, with the following values (average ± standard deviation) for each of the rows of interest, B through G, respectively: 98° ± 1°, 98° ± 1°, 98° ± 2°, 98° ± 3°, 98° ± 4°, and 97° ± 4°. The DC background MF at the control wells was of 4.1 µT (B_horiz_ = 4.0 µT, B_vert_ = 0.6 µT) with a ~95% homogeneity, as measured with the incubator’s door closed.

### 4.3. Flow Cytometry

Analysis of apoptosis was performed by flow cytometry. Immediately after 72 h exposure (see detailed description below), UCBLs were spun down (100 g/10 min), washed with phosphate-buffered saline (PBS, 3.2 mM Na_2_HPO_4_, 0.5 mM KH_2_PO_4_, 1.3 mM KCl, 135 mM NaCl, pH 7.4; reagent grade chemicals were obtained from Sigma-Aldrich (St. Louis, MO, USA), Merck KgaA (Darmstadt, Germany) and Life technologies (Carlsbad, CA, USA)) and resuspended in 100 µL of Annexin kit buffer (Roche, Basel, Switzerland). Cells were then stained with the antibody against the cell surface markers (anti-human CD45-APC clone: 5B1 (isotype: mouse IgG2a) (Miltenyi Biotec, Bergisch Gladbach, Germany)) in order to determine lymphocyte population. Propidium iodide (PI) (BD Biosciences, San Jose, CA, USA) to mark late apoptotic/necrotic (LAN) cells and Annexin-V FITC antibody (BD Biosciences, San Jose, CA, USA) to distinguish the early apoptotic (EA) subpopulation of cells (Annexin-V FITC positive/PI negative cells) was added. Samples were then incubated for 20 min in the dark at room temperature (RT), washed with PBS, spun down, diluted in 200 µL of the Annexin kit buffer and analyzed immediately by the BD Accuri C6 flow cytometer (Accuri Cytometers, Ann Arbor, MI, USA). CD45-APC positive cells were analyzed on the Annexin-V/PI scatter and percentage of the EA and LAN cell populations (Annexin-V FITC positive/PI positive cells) was defined.

### 4.4. Reactive Oxygen Species

After 1 and 3 h lasting exposure the dichlorofluorescein diacetate (DCFDA) solution was added to each sample (final concentration of 10 μM) already pre-treated with CD-45 APC and PI for measuring ROS activity within viable (PI negative) cells. In each experiment, five wells from each row (B–G) at 1 h and five remaining wells at 3 h were pooled together and further processed. A total of 3 independent experiments were carried out. Samples were then incubated for 20 min in the humidified incubator at 37 °C and 5% CO_2_, washed with PBS, spun down, diluted in 200 μL of PBS, and analyzed by flow cytometry immediately.

### 4.5. Statistical Analysis

The one-way ANOVA test was performed for the statistical analysis, followed by the post-hoc Fisher LSD test to compare the exposed groups against the control group. All statistical operations were carried out using Statistica 8.0 software (StatSoft, Tulsa, OK, USA). The results were considered as significantly different at *p* < 0.05.

## Data Availability

Raw data from the figures of this work are available upon request.
